# Probability of informed trading during the COVID-19 pandemic: the case of the Romanian stock market

**DOI:** 10.1186/s40854-022-00415-9

**Published:** 2023-01-15

**Authors:** Cosmin Octavian Cepoi, Victor Dragotă, Ruxandra Trifan, Andreea Iordache

**Affiliations:** 1 Department of Money and Banking and CEFIMO, Faculty of Finance and Banking, Bucharest University of Economic Studies, Bucharest, Romania; 2 Department of Finance and CEFIMO, Faculty of Finance and Banking, Bucharest University of Economic Studies, Bucharest, Romania; 3 Doctoral School of Finance, Faculty of Finance and Banking, Bucharest University of Economic Studies, Bucharest, Romania

**Keywords:** PIN, COVID-19, Market microstructure, Insider trading

## Abstract

Using data from the Bucharest Stock Exchange, we examine the factors influencing the probability of informed trading (PIN) during February—October 2020, a COVID-19 pandemic period. Based on an unconditional quantile regression approach, we show that PIN exhibit asymmetric dependency with liquidity and trading costs. Furthermore, building a customized database that contains all insider transactions on the Bucharest Stock Exchange, we reveal that these types of orders monotonically increase the information asymmetry from the 50th to the 90th quantile throughout the PIN distribution. Finally, we bring strong empirical evidence associating the level of information asymmetry to the level of fake news related to the COVID-19 pandemic. This novel result suggests that during episodes when the level of PIN is medium to high (between 15 and 50%), any COVID-19 related news classified as misinformation released during the lockdown period, is discouraging informed traders to place buy or sell orders conditioned by their private information.

## Introduction

The recent COVID-19 pandemic has led to a quick response of financial markets (Aslam et al. 2020; Espinoza-Mendez and Arias 2021), eroding a quarter of wealth in nearly a month (Ali et al. 2020). The significant impact of the COVID-19 pandemic on stock markets has been studied in an increasing body of literature (Padhan and Prabheesh 2021). Markets have responded in various ways: the coronavirus pandemic has been found to increase market volatility (Albulescu 2021; Ali et al. 2020; Ftiti et al. 2021), trading volume (Chiah and Zhong 2020) and to decrease stock returns (Ashraf 2021). Interestingly, even similarities in the name with COVID-19 have determined a decrease in company stock prices (Corbet et al. 2021).

Questions have been raised about investors' reactions since the outbreak differed from one capital market to another due to various cultural, demographic, and country-specific factors. For example, Fernandez-Perez et al. (2021) have found that cultural aspects are of great importance in the way investors assimilate new information during the COVID-19 pandemic. Specifically, their results have shown larger declines and higher volatility in stock markets within countries characterized by lower individualistic behaviour and higher tendencies to avoid uncertainty, during the first three weeks after the first announcement of the COVID-19 case. Furthermore, Kizys et al. (2021) have found evidence of herding behaviour in the first three months of the COVID-19 outbreak, which has been gradually reduced afterwards by a powerful government response to the pandemic. Bouri et al. (2021) have also studied the effect of the Covid-19 pandemic on investor herding behaviour using a sample of 49 international stock markets and have found solid evidence of herd formation mainly during the Covid-19 outbreak period. The herding behaviour following the pandemic-induced uncertainty has been identified to be more intense in the emerging capital markets, as well as in the Southern European Countries (like Portugal, Italy, Greece, and Spain), whose economies have been amongst the hardest hit by the pandemic.

Rouatbi et al. (2021) have shown that the vaccination process helped stabilize global stock markets, causing a drop in volatility, regardless of the government's policy response. However, the financial markets from the developed countries incorporated the news about the anti-COVID-19 vaccine much faster compared to the emerging ones where the volatility was also higher in the weeks after the announcement of the Pfizer-BioNTech COVID-19 vaccine. In light of the foregoing, our study raises two important research questions. Firstly, has the COVID-19 financial crash changed the way informed traders adjusted their behaviour? Secondly, what are the factors that influence informed traders’ participation in the trading process?

The study of informed trading during financial turmoil is not new (Easley et al, 2011). However, the COVID-19 pandemic is different from other economic crises for several reasons: (1) the existence of lockdowns constraining the economic activity of the agents who could have revived the economy, (2) the psychological effects according to which the fear of contracting the COVID-19 virus discouraged people to engage in some activities, (3) the role of banks, which, during the COVID-19 pandemic, have absorbed the shock.

Moreover, the necessity to determine the likelihood of informed trading, as well as to measure the amount of private information, has undergone a rapid increase in models and empirical studies in the market microstructure literature. Thus, asymmetrical information, its causes and effects, are probably one of the main topics in finance, in very different contexts (Aboody and Lev 2000; Healy and Palepu 2001; Duarte and Young 2009, etc.). Consequently, the probability of informed trading (hereafter PIN) is widely recognized as a proxy for asymmetrical information (Easley et al. 1996, 2002; Aslan et al. 2011; Agudelo et al. 2015, etc.). Therefore, as stated previously, an important direction for study is the identification of the appropriate factors that could have an impact on PIN (Aslan et al. 2011; Sankaraguruswamy et al. 2013; Agudelo et al. 2015, etc.). This concern is present in studies on developed capital markets (Aslan et al. 2011; Sankaraguruswamy et al. 2013), but also on some emerging ones (Agudelo et al. 2015).

The Romanian capital market has been studied extensively due to its particularities. Among its specificities, which confer interest, are the low trading volume and liquidity (Dragotă and Mitrică 2004; Filip and Raffournier 2010), a large state ownership among the listed firms (Pop 2006), a lower degree of market efficiency (Dragotă and Țilică 2014), and a low financial information coverage (measured through the analysts/firm ratio) (Albu et al. 2021). Furthermore, Albu et al. (2021) have found that senior executives extract abnormal returns. However, herding behaviour has not been identified in the Romanian capital market (Pochea et al. 2017).

Investigating the level of information asymmetry is not new for the Romanian capital market (Cepoi and Toma 2016). However, the present analysis is focused on identifying the determinants of PIN, in the pandemic context, which is a period of increased uncertainty, with rapid and significant crashes followed by consistent recoveries and periods of stagnation. We expect that changes might occur in this new environment and some novel stylized facts regarding information asymmetry to come to light. We present a new contribution to the study of PIN, considering insider transactions, for which data on transactions performed by this type of traders are disclosed within the Bucharest Stock Exchange.^1^

We provide new insights on the driving factors and the dynamic of information asymmetry by employing a methodology that accounts for the distributional characteristics of the PIN. Specifically, we use an unconditional quantile regression model for panel data to examine the effects of a series of variables that describe trading activity, volatility, or news related to the COVID-19 pandemic at different intervals throughout the PIN distribution. Compared with other standard mean regression frameworks, the quantile regression model has the capacity to draw inferences regarding the observations that rank above or below the information asymmetry conditional mean (Koenker and Bassett 1978). Since it does not require any specific hypothesis about the distribution of error terms, the sensitivity to outliers is less significant in comparison to mean regression, so it can provide more accurate and robust regression results. We apply this procedure to the case of the Romanian capital market. Compared to other studies that use classical regression, some previous studies investigating investor behaviour on the Bucharest Stock Exchange have provided some interesting features under a quantile regression framework (Pochea et al. 2017; Toma et al. 2021). The overconfidence among Romanian investors has a greater positive effect on higher returns for shorter investment horizons—lower than one year (Toma et al. 2021). Therefore, considering that our paper deals with investors’ behaviour, during a period characterized by fear and low overconfidence (COVID-19 pandemic), the quantile regression framework may be the best approach for conducting estimates.

This study extends the literature by exploring the impact of the COVID-19 pandemic on information asymmetry, measured through PIN for the case of an emerging capital market, respectively, the Romanian one, given the COVID-19 situation. We have considered the period between February 2020 and October 2020. This time window includes a period with normal market conditions (1 February 2020 to 15 March 2020), a lockdown period (16 March 2020 to 14 May 2020), and a period of revival of the Romanian capital market (15 May to 1 October 2020). Based on an unconditional quantile regression approach, we show that PIN exhibits asymmetric dependency with liquidity and trading costs. Furthermore, by building a customized database that contains all insider transactions on the Bucharest Stock Exchange, we reveal that these types of orders monotonically increase the information asymmetry from the 50th to the 90th quantile throughout the PIN distribution. Finally, we bring forth strong empirical evidence associating the level of information asymmetry with the level of fake news related to the COVID-19 pandemic. This novel result suggests that during episodes when the level of PIN is medium to high (between 15 and 50%), any COVID-19 related news classified as misinformation released during the lockdown period is discouraging informed traders to place buy or sell orders conditioned by their private information.

Our paper may be useful to academics, investors, and regulators. A single country analysis can provide a deeper understanding of a financial phenomenon, as long as substantial differences from one market to another, even for apparently similar cases, are noticed in different studies (Filip and Raffournier 2010; Dragotă and Țilică 2014; Albu et al. 2021). Even if all markets are affected by the same shock, their reaction can be different. Our study might be useful for investors, who are interested in the level of informational asymmetry in order not to carry out transactions with higher costs, and regulators, who want to reduce the informational asymmetry. For example, investors are interested in knowing the level of information asymmetry associated with a certain market, since a higher level of this metric translates into higher trading costs, lower transparency, and implicitly into fewer efficient decisions on asset allocation. Furthermore, regulators are interested in diminishing the level of information asymmetry, especially in the situation of cross-listed stocks, since naïve traders, when observing such a phenomenon, can choose other trading platforms to execute their orders. Finally, all of these issues can be useful to portfolio managers, especially in the context of international diversification.

The remainder of the paper is structured as follows. In “Background and hypothesis tested” section, the theoretical background is presented and the tested hypotheses are proposed. In “Econometric approach” section, the econometric approach (research design) is presented. In “Data description” section data and institutional background are described. Section Results” section discusses the results. Section Conclusions” concludes.

## Background and hypothesis tested

### Theoretical background of PIN

In the market microstructure literature, asymmetric information is approached from different perspectives (Easley et al. 1996; Aboody and Lev 2000; Healy and Palepu 2001; Duarte and Young 2009). Regarding the information asymmetry in capital markets, one direction of analysis is focused on informed trading (Easley et al. 1996, 2002, 2008). Informed trading is estimated through different measures: probability of informed trading (see, e.g., Easley et al. 1996, 2002, 2008; Kang 2010; Aslan et al. 2011; Agudelo et al. 2015), probability of medium-sized contrarian trades (Chang and Wang 2019), adverse selection component of the bid-ask spread^2^ (Borisova and Yadav 2015).

Among these different measures, the probability of informed trading is widely used in the literature as a proxy for information asymmetry (e.g., Easley et al. 1996, 2002, 2008; Aslan et al. 2011; Agudelo et al. 2015). PIN refers to “*the probability that any trade that occurs at time t is information-based*” (Easley et al. 1996). Basically, PIN is a good measure of the level of private information embodied in prices. When the PIN is higher (lower), it means that a larger (smaller) fraction of orders is based on private information, hence stock prices contain a greater (fewer) amount of private information from investors (Xu 2021). Private information refers to news, statistics, or data on the results, strategies, or plans of a firm that are available for free to some employees, managers, or authorities, but that can be accessible only for a cost to the other agents.^3^

### Estimating PIN

Estimating the level of informational asymmetry between different types of investors can be historically connected with the strong form of market efficiency (Fama 1970). The seminal papers of Easley and O’Hara (1987, 1992) and Easley et al. (1996) describe the model. They are considering trading as a competition between market markers, i.e., the liquidity providers and different types of traders taking place over several trading days. Orders arise from informed or uninformed (naive) traders. Regardless of the trading day, the arrival of buy and sell orders coming from naive traders is shaped according to two independent Poisson processes, with daily arrival rates ${\epsilon }_{B}$ and ${\epsilon }_{S}$, respectively. Informational events occur between trading days with probability $\alpha$. Consequently, the probability that a certain day will be information neutral is $(1-\alpha$). Informed agents trade only on days with information events, buying during days with good news (which appear with probability $1-\gamma$) and selling during days with bad news (which appear with probability γ). The orders submitted by informed traders follow a Poisson process with a daily arrival rate $\mu$.

**Fig. 1:**
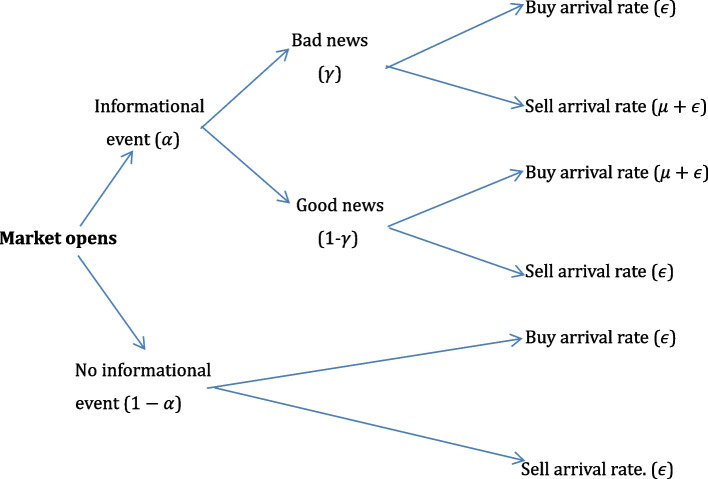
Derivation of the PIN, Easley et al. (1996)

The derivation of PIN is presented in Fig. 1. To simplify the analysis, we assume, in accordance with the recommendation of Easley et al. (1996), that ${\epsilon }_{B}={\epsilon }_{S}=\epsilon$.

After the estimation of the parameters, the degree of information asymmetry given by the PIN has the following formula (Easley et al. 1996): $PIN=\alpha\mu/\left(\alpha\mu+2\epsilon\right)$. Although it is a widespread usage among researchers, the initial model proposed by Easley and O'Hara (1992) may provide biased results (Yan and Zhang 2012). To overcome these biases, several studies present different approaches for PIN estimation (Easley et al. 2008, 2010; Yan and Zhang 2012; Gan et al. 2015).

Most of the papers estimate PIN over long periods.^4^ The capital markets usually respond to news almost instantly (Aslam et al. 2020; Espinoza-Mendez and Arias 2021); therefore, the computation of PIN over long periods represents a shortcoming (Pöppe et al. 2016). Few articles in the existing literature analyse PIN over short periods (Tay et al. 2009; Pöppe et al. 2016). The disadvantage of the estimation for a longer period is acknowledged by Easley et al. (2008).

### Determinants of PIN in the market microstructure literature

The market microstructure literature has documented that PIN is sensitive to different factors (Tang et al. 2010; Chen and Choi 2012; Sankaraguruswamy et al. 2013). We have included in Table 1 only the studies in which static PIN^5^ has been considered as the dependent variable, in absolute value, as in Aslan et al. (2011) or as logarithm, as in Sankaraguruswamy et al. (2013). In addition, Agudelo et al. (2015) use the daily change of dynamic PIN as a dependent variable.

**Table 1:** Studies on the determinants of PIN

Factor	Definition	Studies/expected sign	Observations
PIN (t − 1)	PIN estimated for the previous period	Chen et al. (2007) (+)	The autoregressive framework is preferred to control for the persistence in time of information asymmetry
Spread	$\frac{ask-bid}{(ask+bid)/2}$	Chen and Choi (2012) (+), Ahern (2020) (+)	The bid-ask spread is divided by mid-quotes to measure the relative discrepancy between the bid and ask quotes
Trading volume	The average of the logarithms of the total daily trading volumes computed for each month	Chen and Choi (2012) (+), Sankaraguruswamy et al. (2013) (−)	Agudelo et al. (2015) (−) use the number of trades as the independent variable. Sankaraguruswamy et al. (2013) (−) use price as an independent variable
Market capitalization	The logarithm of the market capitalization for each company	Tang et al. (2010) (+), Zhang and Yan (2015) (−), Aslan et al. (2011) (−), Sankaraguruswamy et al. (2013) (−)	
Volatility	The standard deviation of daily stock returns	Sankaraguruswamy et al. (2013) (−)	

Recently, Ahern (2020) has used real illegal insider trading records to investigate the major drivers of information asymmetry and has found that only the order imbalance and the spread can be considered robust drivers for the propensity to execute informed trades.

Other interesting variables have also been included in other studies, but unfortunately the data architecture does not allow their calculation. More specifically, Chen and Choi (2012) have linked the probability of informed trading to the price discovery process, proxied by Harsbrouck's information share. However, the companies selected for this study are not cross-listed, and price discovery was not possible in this situation. Thus, we account for all market microstructure aspects by including the spread, realized volatility, and trading volume.

### Hypothesis development

In line with the purpose of our paper, we have investigated the impact of the COVID-19 event on PIN. Different measures can be used to test this hypothesis. Thus, the emergence of COVID-19 by the first reported case (Espinoza-Mendez and Arias 2021) or statistics regarding the number of new cases, the number of new deaths, and the number of patients in intensive care medical units, all related to COVID-19, can influence the perception of investors (homo sapiens, after all) (see the senses of homo sapiens and homo economicus in Thaler 2000) regarding the future of their investments on stock exchanges. Moreover, Bouri et al. (2021) have emphasized the pandemic’s role in shaping behavioural patterns in the financial markets. Also, the impact of different regulations can induce a sentiment of certainty or, on the contrary, of uncertainty. RavenPack platform provides some indicators related to COVID^6^ such as Panic Index, Media Coverage Index, Infodemic Index, Country Sentiment Index, and Media Hype Index. Some of these indexes, Panic Index, Media Coverage Index, Infodemic Index, Fake News Index, and Media Hype Index, are related to news regarding COVID-19. Some of them are related to panic, hysteria, or sentiment across all entities mentioned in the news, e.g., Panic Index or Country Sentiment Index. Testing the impact of these indexes on PIN can take into account the diversity of issues related to COVID-19, a complex reality that amalgamates medical and political measures, reactions of the population, etc. We have considered, based on correlation coefficients and statistical significance, three of these indexes in our study to test different dimensions of the implications of COVID-19 on investor behaviour (Fake News Index, Media Coverage Index, and Infodemic Index).

Two extreme reactions of investors to an event like COVID-19 can be considered. According to the first possible reaction, investors (in their quality of homo economicus) should try to benefit from the periods of panic manifested on the stock exchanges during crisis times, like the COVID-19 pandemic. They should try to practice active portfolio management and earn systematic abnormal earnings, using different strategies (Albu et al. 2021), including benefiting from a higher PIN. In this case, we can expect the indicators for COVID-19 related news and sentiments to cause an increase in PIN. Thus, the first hypothesis tested is:

#### H1

PIN increases in the COVID-19 period.

According to a second possible reaction, investors (in their quality of homo sapiens) (Thaler 2000; Bloomfield 2010; Lo and Zhang 2018; Lo 2019) should prefer to be cohesive with other individuals, in order to maximize their utility function (Statman 2019). It can be argued that this approach is not in line with the classical literature on market microstructure, but it is in line with behavioural finance (Statman 2019). It can be noticed that the COVID-19 crisis was associated with donations from different people who participated in the fight against COVID-19. This behaviour is coherent if the expected return of the investment is a function of wants for utilitarian benefits, but also wants for expressive and emotional benefits (Statman 2019). From this perspective, in the COVID-19 period, the PIN should be lower. In this case, we can expect that the proxy for news and sentiments related to COVID determines a decrease in PIN. Thus, the alternative hypothesis tested is:

#### AH1

PIN decreases in the COVID-19 period.

Investor behaviour should be influenced by the presence of uncertainty amplified by the number of news related to the COVID-19 pandemic. This uncertainty is determined by epidemiological evolutions, but also by the authorities’ decisions (Nagar et al. 2019). Additionally, previous studies showed that an increase in economic uncertainty leads to an increase in spread and other information asymmetry proxies (Nagar et al. 2019). Therefore, the number of news sources covering the topic, the number of fake news about the COVID-19 pandemic, and all other indicators chosen could modify the investor's behaviour, allowing traders who have more information to increase their gains. In addition, the news on the pandemic could lead to more information asymmetry between investors. Thus, the second tested hypothesis is as follows:

#### H2

Sentiment related to COVID-19 determines an increase in PIN.

However, even informed traders could be taken by surprise in a pandemic and modify their behaviour in a sense of irrationality or misjudgement. Analysing the insider trading on the Bucharest Stock Exchange, Albu et al. (2021) find such results corresponding to the global financial crisis period (2007–2009). Thus, the spread, in terms of information interpretation, between informed and naive traders should be reduced, also causing a decrease in PIN. Consequently, the alternative second tested hypothesis is:

#### AH2

Sentiment-related to COVID-19 determine a decrease in PIN.

In addition, to test the two hypotheses together, we also used an interaction between the lockdown period and the related variables of COVID-19. This is a mandatory approach since the uncertainty regarding the evolution of the COVID-19 virus and the vaccine was extremely high.

Last but not least, we have introduced a new factor in our analysis, namely the insiders’ transactions, in order to test its relationship with PIN. We have built a database with all insider transactions reported to the Bucharest Stock Exchange. From this perspective, we were able to directly test whether these insider transactions have had an impact on information asymmetry (PIN).

The relationship between information asymmetry and insider trading has been analyzed by several researchers (see, for example, Grossman and Stiglitz 1980; Kyle 1985; Huddart and Ke 2007). Both models of Grossman and Stiglitz (1980) and Kyle (1985) emphasized that a higher level of information asymmetry contributes to significant abnormal returns generated by insiders’ trades (higher abnormal returns associated with purchases, lower (negative) abnormal returns associated with sales) and greater profits. However, for the relationship between the insiders’ trade volume and the information asymmetry, the evidence is different. While the model of Grossman and Stiglitz (1980) highlights a positive relationship between the insiders’ trade volume and the information asymmetry, Kyle (1985) found no relationship between the two. Huddart and Ke (2007) extended the analysis and found similar results to Grossman and Stiglitz (1980) between the information asymmetry (measured through 3 proxies, namely the book-to-market ratio, research and development expenditures within a firm and the median absolute abnormal return following past earnings announcements) and insiders’ trades’ volume.

Since insider transactions may be performed based on liquidity or portfolio rebalancing reasons, a significant proportion of trades, though, can be instructed by these insiders on informational advantage with respect to the prospects of the firm compared to other market agents—who do not have the same access to value-relevant information of the firm (Huddart and Ke 2007; Albu et al. 2021). Therefore, a higher volume of insider trades or a higher frequency of these insider trades could be associated with a higher level of information asymmetry. Thus, the third hypothesis tested is:

#### H3

Insider transactions determine an increase in PIN.

## Econometric approach

The purpose of the present analysis is to identify the factors that influence the level of information asymmetry on the Bucharest Stock Exchange during the COVID-19 pandemic. Our model is specified as follows:

1 $${PIN}_{i,t}={\alpha }_{i}+{{\beta }_{1}PIN}_{i,t-1}+{{\beta }_{2}IT}_{i,t}+{{\beta }_{3}TRV}_{i,t}+{{\beta }_{4}CRV}_{t}+{\beta }_{5}*{INT}_{t}+{\varepsilon }_{i,t}.$$

In Eq. (1), $i=\stackrel{-}{1,N}$ and $t=\stackrel{-}{1,T}$, are firms and weeks respectively, ${PIN}_{i,t}$ is the probability of informed trading developed by Easley et al. (1996, 2002) and represents a proxy for information asymmetry. Moreover, ${IT}_{i,t}$ quantifies the number of insiders’ transactions, ${TRV}_{i,t}$ is a matrix of trade related variables such as spread, traded volume, market capitalization, or volatility, while ${CRV}_{t}$ includes a series of pandemic related information. ${INT}_{t}$ captures the impact exhibited by COVID-19 related information such as Fake News, Media Coverage, and Infodemic Index on PIN during the lockdown period, which was a period with increased uncertainty, and extremely surprising evolutions on financial markets. Finally, ${\varepsilon }_{i,t}$ represents the error term.

To address the endogeneity issues in Eq. (1) when dealing with the causal effects of various measures of market quality on information asymmetry, Frijns et al. (2015) proposed a panel GMM approach. However, in some cases, mean regression such as OLS or GMM may provide an incomplete image when investigating the link between information asymmetry and other factors, especially during periods of financial crisis (du Plooy 2019). More to the point, a less informed investor can be interested more in what factors or events are amplifying or decreasing the information asymmetry when its level is already high/low, rather than factors that explain the PIN’s dynamic on average.

To overcome the aforementioned limitation, a useful approach is to implement quantile regression. This method, proposed by Koenker and Bassett (1978) has the capacity to draw inferences regarding the observations that rank above or below the information asymmetry conditional mean. Since it does not have any specific hypothesis about the distribution of error terms, the sensitivity to outliers is less significant in comparison to the mean regression, so it can provide more accurate and robust regression results. Given its features and advantages, quantile regression has become a useful tool in financial studies during the last decade (Giglio et al. 2016; Baruník and Čech 2021; Galán 2020).

Generally, for any level $\tau$, across PIN’s conditional distribution denoted $y$, given the set of explanatory variables specified in Eq. (6) and denoted $x$, the conditional quantile ${Q}_{y}\left(\tau |x\right)$ shows $inf\left\{k:C\left(k|x\right)\ge \tau \right\}$ where $C\left(*|x\right)$ represents the conditional distribution function. To evaluate the impact of a certain factor or event at a particular position throughout the PIN distribution, the most common approach given our data structure is the conditional quantile regression (CQR) for panel data developed by Koenker (2004):

2 $${Q}_{{y}_{i,t}}\left(\tau |{x}_{i,t}\right)={\alpha }_{i}+{x}_{i,t}^{T}{\beta }^{CQR}\left(\tau \right).$$

In Eq. (2) ${y}_{i,t}$ is the firm-related information asymmetry, ${x}_{i,t}$ denotes the set of explanatory variables, ${\beta }^{CQR}\left(\tau \right)$ is the common slope coefficient, while ${\alpha }_{i}$ is a location shift coefficient on the conditional quantile of the response. To control for the unobserved firm heterogeneity, Koenker (2004) treats the fixed effects as nuisance parameters. The inventiveness of this approach lies in the introduction of a penalty factor in the minimization algorithm leading to:

3 $$\underset{\left(\alpha ,\beta \right)}{\mathit{min}}\sum_{k=1}^{K}\sum_{t=1}^{T}\sum_{i=1}^{N}{w}_{k}{\rho }_{{\tau }_{k}}\left({y}_{i,t}-{\alpha }_{i}-{x}_{i,t}^{T}\beta \left({\tau }_{k}\right)\right)+\lambda \sum_{i}^{N}\left|{\alpha }_{i}\right|.$$

In Eq. (3), $K$ is the quantiles’ index, ${\rho }_{{\tau }_{k}}$ represents the quantile loss function while ${w}_{k}$ is the relative weight associated with the $k$th quantile. The penalty term $\lambda$ is introduced to diminish the individual effects to zero, leading to an improvement in the quality of the estimates of $\beta$. Furthermore, when λ approaches zero, the model converges to a standard specification with fixed effects. In contrast, the representation given by Eq. (3) becomes a panel model without individual effects when $\uplambda \to \infty$.

However, as Dong et al. (2020) argued, in the CQR the dependent variable distribution is specified conditional on a certain set of factors, leading to a serious limitation, since it cannot capture the dependence structures in its entirety. To overcome this issue, Firpo et al. (2009) developed unconditional quantile regression (UQR) by using the influence function (IF) and the recentred influence function (RIF). More exactly, the IF is an analytical method quantifying the influence of a particular factor on a distributional statistic and has the following form:

4 $$IF\left({y}_{i,t};v\left({F}_{{y}_{i,t}}\right)\right)=\underset{\varepsilon \to 0}{\mathrm{lim}}\left(\frac{v\left[\left(1-\varepsilon \right){F}_{{y}_{i,t}}+\varepsilon {G}_{{y}_{i,t}}\right]-v\left({F}_{{y}_{i,t}}\right)}{\varepsilon }\right).$$

In Eq. (4),$0\le \varepsilon \le 1$, ${F}_{{y}_{i,t}}$ represents the cumulative distribution function of ${y}_{i,t}$, ${G}_{{y}_{i,t}}$ denotes the distribution that puts mass at the value ${y}_{i,t}$, while $v\left({F}_{{y}_{i,t}}\right)$ is the value of the statistic. The RIF is an estimator $\nu$ with a probability distribution $F$ at point ${y}_{i,t}$ and is computed by adding this statistic to its IF:

5 $$RIF\left({y}_{i,t};v\left({F}_{{y}_{i,t}}\right)\right)=v\left({F}_{{y}_{i,t}}\right)+IF\left({y}_{i,t};v\left({F}_{{y}_{i,t}}\right)\right).$$

In Eq. (5), the expected value of the RIF is $v\left({F}_{y}\right)$, as long as the expected value of the $IF\left({y}_{i,t};v\left({F}_{{y}_{i,t}}\right)\right)$ is zero. This suggests that regressing a particular statistic, such as the mean, generates the same coefficients as the OLS estimates, and this principle applies to any statistics of interest along the dependent variable distribution. Furthermore, according to Dong et al. (2020), the conditional expectation of the $RIF\left(y;v\left({F}_{y}\right)\right)$ can be designed as a function of the explanatory variables, i.e.,$E\left[RIF\left({y}_{i,t};v\left({F}_{{y}_{i,t}}\right)\right)|{x}_{i,t}\right]={m}_{v}\left({x}_{i,t}\right)$. In addition, if we select the τth quantile as the statistic of interest and choose to estimate the density functions for each quantile based on Kernel density techniques, the RIF, given ${q}_{\tau }$ is specified as follows:

6 $$RIF\left({y}_{i,t};{q}_{\tau };{F}_{{y}_{i,t}}\right)={q}_{\tau }+IF\left({y}_{i,t};{q}_{\tau };{F}_{{y}_{i,t}}\right)={q}_{\tau }+\frac{\tau -{\mathbb{I}}\left\{{y}_{i,t}\le {q}_{\tau }\right\}}{{f}_{{y}_{i,t}}\left({q}_{\tau }\right)}.$$

In Eq. (6), ${q}_{\tau }$ represents the τth quantile of the unconditional distribution of the information asymmetry ${y}_{i,t}$, ${f}_{{y}_{i,t}}\left({q}_{\tau }\right)$ express the probability density function of ${y}_{i,t}$ evaluated at the τth quantile based, while ${\mathbb{I}}\left\{{y}_{i,t}\le {q}_{\tau }\right\}$ is an indicator function showing whether ${y}_{i,t}$ falls below the τth quantile or otherwise. Thus, the UQR estimator can be seen as the coefficient,${\beta }^{UQR}\left(\tau \right)$, of the RIF given the explanatory variables:

7 $$RIF\left({y}_{i,t};{q}_{\tau };{F}_{{y}_{i,t}}\right)={x}_{i,t}^{T}{\beta }^{UQR}\left(\tau \right).$$

Considering the aforementioned arguments, we adopt as the baseline specification the unconditional quantile regression (UQR) for panel data developed by Borgen (2016), which accounts for high-dimensional fixed effects.

## Data description

### Investigating PIN on Bucharest Stock Exchange

**Fig. 2:**
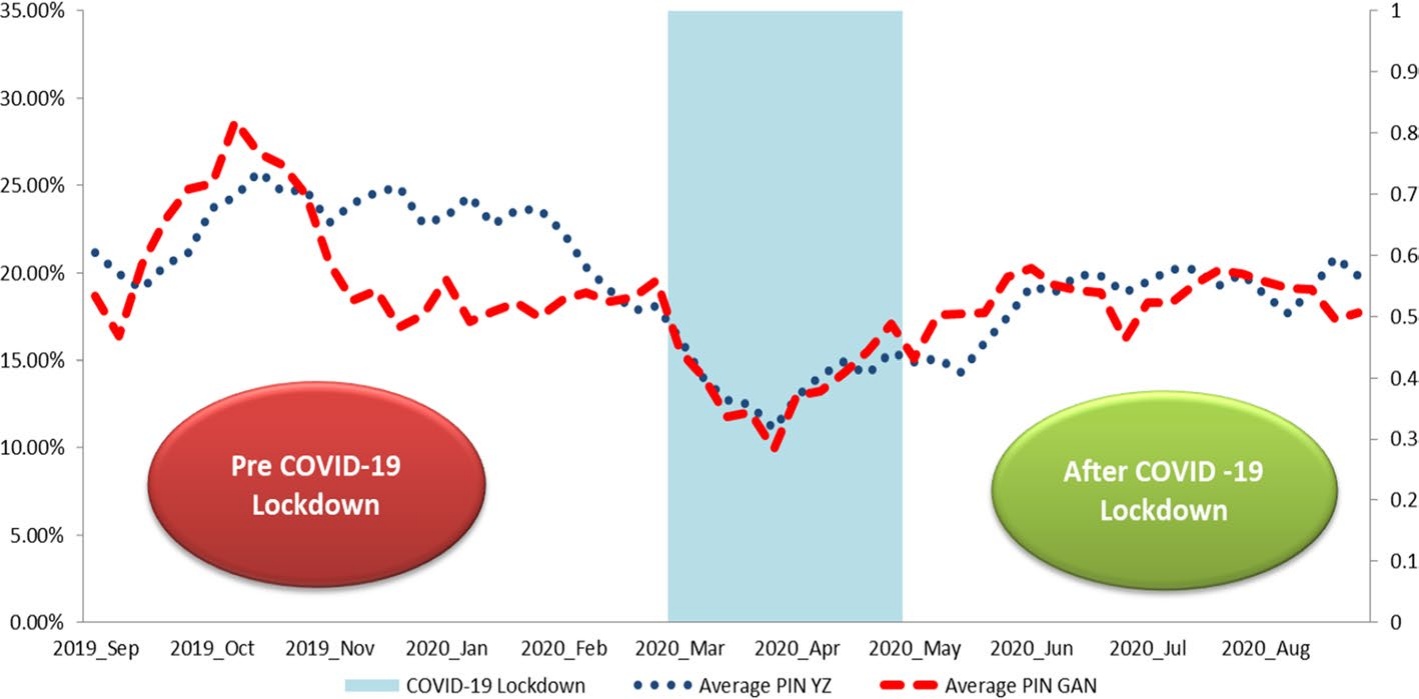
Average PIN: YZ (Yan and Zhang 2012) versus GAN (Gan et al. 2015)

To investigate the factors influencing information asymmetry, we rely on 12 companies listed on the Bucharest Stock Exchange (BSE) from February 2020 to October 2020. The selection of shares was constrained by the lack of liquidity in the market. However, this portfolio is representative of the Romanian capital market and is diversified, including companies from key sectors (like financial, energy and utility, healthcare), covering more than 50% of the market capitalization. We extract from Bloomberg 1 min tick data regarding the best ask, the best bid, and the associated volumes. The trade direction was computed using the Lee and Ready (1991) algorithm.^7^ We exclude from the analysis the trades conducted in the first and last 15 min of the trading sessions as recommended by Pöppe et al. (2016).^8^ To estimate the weekly^9^ PIN, we use the approach proposed by Yan and Zhang (2012)^10^ and Gan et al. (2015)^11^ respectively. The results are presented in Fig. 2:

As we can see in Fig. 2, both measures^12^ experienced a substantial decrease during the COVID-19 lockdown compared to the previous period,^13^ followed by a moderate recovery in the next months. Indeed, during pre COVID-19 pandemic, the average value for PIN, according to Yan and Zhang (2012) was 22.7% and 20.45% according to Gan et al. (2015). During the lockdown period, the average PIN was 13.80% and 13.65% respectively. After the lifting of travel restrictions by the Romanian government, which also coincided with the revival of the capital market the average PIN was 18.47% and 18.51% according to Yan and Zhang (2012) and Gan et al. (2015). Furthermore, we test for five structural breaks in both time series presented in Fig. 2. For the PIN computed using Yan and Zhang (2012) procedure, the Bai and Perron (2003) test reported the last week of February 2020 as a breakpoint while for the PIN computed using Gan et al. (2015) we identified the first week of March 2020 as a breakpoint.

This implies that the increase in uncertainty due to the pandemic in the Romanian capital market has led to a decrease in information asymmetry during periods of bear market. Moreover, during periods of sustained recovery, the information asymmetry measured by PIN experienced smooth and gradual increases. These empirical facts urge for a more thorough investigation regarding the factors that contributed to this dynamic of PIN. We control for this aspect by considering the interactions between the lockdown period and COVID-19 related variables.

The average PIN for all companies included in the study is around 18% according to the Yan and Zhang (2012) estimation algorithm and 17% according to Gan et al. (2015). These values are similar to those reported by Kang (2010) and Aslan et al. (2011) for more advanced countries. Compared with other studies such as Easley et al. (1996) or Pöppe et al. (2016), we fail to identify a clear pattern suggesting that PIN is strongly decreasing from low to high volume stocks.

**Fig. 3:**
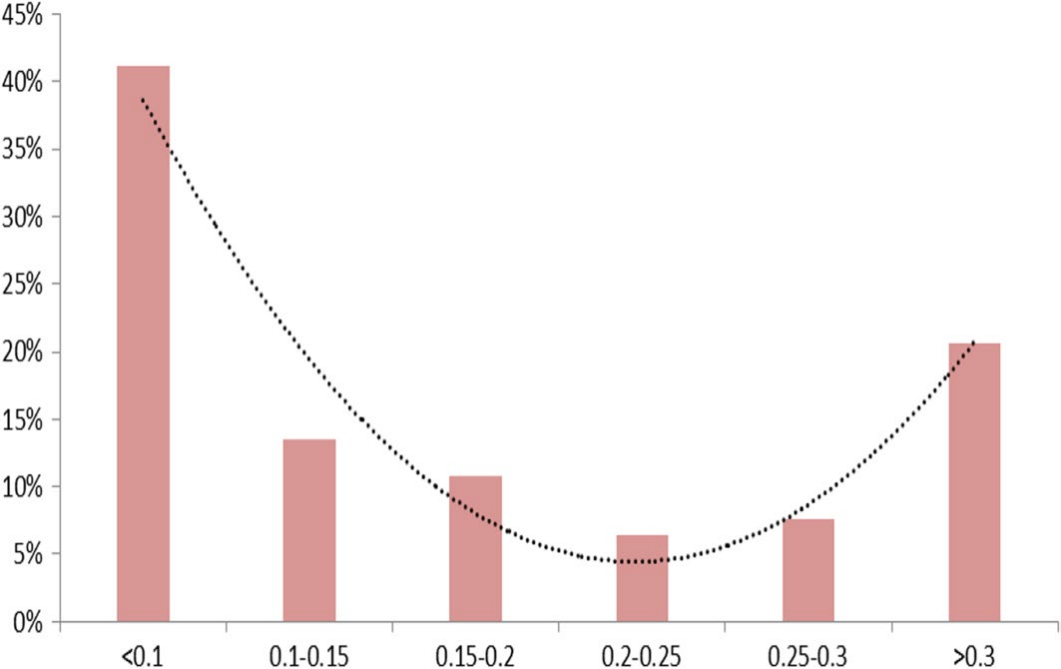
Distribution of PIN

To have a more comprehensive view of the distribution of PIN, we present the histogram in Fig. 3 alongside a polynomial trend line. The results clearly suggest that explaining the factors influencing the level of PIN during the COVID-19 pandemic could provide some misleading results when using a linear approach such as Panel Fixed Effects or Panel GMM.

### The explanatory variables

To account for the impact induced by the novel pandemic, we include, alongside the control variables, three additional measures related to COVID-19 extracted from the RavenPack analytics tool. This platform provides real-time media analytics and statistics, related to the Coronavirus pandemic and various topics from corporate finance to financial markets. It covers sources such as Dow Jones Newswire, Wallstreet Journal, and StockTwits among others (Haroon and Rizvi 2020; Umar et al. 2021).

In addition, we include a novel factor that captures the insider’s activity on the Bucharest Stock Exchange. Insider trading has been largely covered by academics lately (Seyhun 1986, 1988; Lakonishok and Lee 2001; Jeng et al. 2003; Hillier et al. 2015), and the consensus is that insiders have better information than regular traders do. Any cross-sectional differences in the informational advantage that insiders have (which are reflected in their proneness to trade) can also be related to the PIN. Although the insider transactions-to-total transactions ratio is lower than PIN regardless of the selected company, both are likely driven by the features of the firm, therefore being positively correlated.

The data gathered includes insider identification details and trade specifics. Insider identification covers the insider’s type (company or individual), name, and position within the company, while trade specifics refer to transaction date, type (acquisition, sale, or share buyback), underlying stock, volume, and price. An insider is defined by the Bucharest Stock Exchange as “any agent that holds a leadership position (such as administrative, management, and control bodies) within a listed company, as well as those with whom she/he has a close relationship (spouse, children, relatives, etc.) or with whom she/he acts in concert”.^14^ All insiders are required to report to the Financial Supervisory Authority (ASF) all trades performed in their account related to the issuer’s shares with whom they are considered insiders. The notification has to be made no longer than 5 working days from the transaction date. In addition, the intermediary through whom the trades are booked has to disclose them to the market operator as soon as possible, to allow the market to reveal the information before the next trading session.

Apart from any corporate insiders’ trades, there are also reported share buyback programs by the issuing companies, which represent approx. 80% of all insider trades reported within the analysed timeframe and accounted for approx. 88% of all trades volume. In general, these operations have a certain period in which they take place, with the issuer being able to repurchase only a maximum of 10% out of the total number of shares. The price in these cases is flexible and allows the company's management to buy the necessary package of shares within the established term.

There can be multiple reasons behind these share buybacks. The management of a company considers that the price is undervalued and performs a share buyback program, thus reducing the outstanding number of shares on the market, increasing the percentage of shares owned by the firm's investors (Babenko et al. 2012; Gan et al. 2017). In addition, share buybacks also have a significant impact on earnings per share (Farrell et al. 2014; Almeida et al. 2016). Practitioners and academics alike consider the undervaluation hypothesis as one of the most important reasons for share buybacks (Vermaelen 1981; Ikenberry et al. 1995; Chan et al. 2004; Brav et al. 2005; Liang 2012).

However, managers can profit from the undervaluation of the stock. Companies can repurchase their shares, even in situations where dividends can better serve the shareholders of the company. As a result, this could generate a reduction in the aggregate shareholder value.

Also, a company can opt for a share buyback program for compensation purposes (there are 10 out of 146 trades within our data sample representing stock allocations to managers through stock option plans from February 2020 to October 2020) (Fenn and Liang 2001; Kahle 2002). For example, management and employees are rewarded based on their performance, as well as loyalty programs, triggering a positive outcome with respect to the convergence of managers-shareholder interests. Given that a share buyback is a well-known form of corporate pay-out, the fact that the management has its shares or stock options in the company, can bring together the interests of the management with the ones of the outside shareholders (Brown et al. 2007). In this case, managers would be better motivated to improve the financial performance of the company (Ndayisaba and Ahmed 2021), which would ultimately lead to an increased market value. This compensation form is occasionally used within the listed companies on the Romanian stock market.

In addition, other potential reasons for which companies can opt for share buybacks are: (1) preventing some shareholders from taking a controlling stake in the company (Billet and Xue 2007); (2) distributing cash to shareholders in a more cost–effective and tax-efficient way compared to the distribution of dividends, therefore being a desirable option for shareholders (Rau and Vermaelen 2002); etc. It is important to mention, though, that 40 out of the 83 listed companies on the Romanian regulated market have paid dividends in the 2020 fiscal year, respectively 10 companies from our data sample out of these 40, so we can state that the share buybacks are not an option taken into account by the locally listed companies to the detriment of dividends.

Using as filter our analysed time horizon and the 12 stocks that define our data sample, 146 insider transactions were reported to the Bucharest Stock Exchange. Some descriptive statistics are presented in Table 2.

**Table 2:** Summary of insider trades

Trade type	No. of companies	Trades	Volume (no. of shares)	Total volume (M. Ron)
Purchase trades	1	5	3000	0.079
Sale trades	3	15	4,873,737	27.19
Stock allocation	1	10	13,303	0
Share buybacks	3	116	35,129,750	89.04
All trades	4	146	40,019,790	116.31

During the selected period, only 4 of the 12 firms experienced such transactions. Furthermore, the largest part of the data sample (116 trades out of the 146) is made up of corporate transactions (all share buybacks), the remaining 30 transactions being instructed by individuals, especially current and former managers within the company. This is not surprising. Firstly, in terms of the total number of trades, there are fewer during the analysed period than in previous years, mainly due to pandemics, lockdown, and restrictive measures that followed since the beginning of the spring of 2020, but also due to their effects on businesses and the entire local economy. The activity of companies has been limited and changes were made concerning the handling of their daily activities, initial forecasts have been re-examined, various financing programs were taken into account, etc. Secondly, when looking at the individual transactions being instructed by management, these may be due to their informational advantage over the other insiders/outsiders, having privileged access to confidential or valuable information regarding the current situation of their company, in terms of both financial and operational perspectives, as well as its future prospects (Seyhun 1986; Ke et al. 2003; Goergen et al. 2019).

A detailed description of the variables and their sources is presented in Table 3, while two descriptive statistics tables are presented in Appendices 2 and 3.

**Table 3:** Description of the variables

Name	Symbol	Description
Probability of Informed Trading	PIN	Computed weekly for each company following Yan and Zhang (2012) and Gan et al. (2015). We have split the trading day into intervals as in Pöppe et al. (2016)
Quoted Spread	SQ	The quoted or absolute spread measures the difference between the best ask price and the best bid price. As an observation, the relative spread (used by Chen and Choi 2012) and the absolute spread are highly correlated (higher than 99%). (Source: own computation)
The Logarithm of Weekly Volume	LWAV	We compute the average of the daily number of shares traded within a week. Due to the size of the variable, we consider the logarithm of the weekly volume. (Source: own computation)
The Logarithm of Weekly Market Capitalisation	LWMC	We compute the weekly market capitalization on a firm level. Following Aslan et al. (2011), we consider the logarithm of the weekly market value. (Source: own computation)
Realized Volatility	RV	$\mathrm{Realized volatility is computed as}: rCo{v}_{t}={\sum }_{i=1}^{M}{r}_{t,i}{r}_{t,i}^{^{\prime}}$ where r_t,i_ is a return vector and $i=1,.., M$ is the number of intraday returns. (Source: own computation)
The Media Coverage Index	MCI	It shows the percentage of all news sources covering the topic of the novel coronavirus in total news sources covered by RavenPack. Values range between 0 and 100, where a value of 50.00 means that 50% of all sampled news providers are currently covering stories about COVID-19. (Source: https://coronavirus.ravenpack.com/)
The Infodemic Index	INF	It calculates the percentage of all entities such as places, companies, etc. that are somehow associated with COVID-19. Values range between 0 and 100, where a value of 50.00 means that 50% of all entities covered by the media are being associated or co-mentioned with COVID-19. (Source: https://coronavirus.ravenpack.com/)
The Fake News Index	FNI	It measures the level of media chatter about the novel pandemic that refers to misinformation alongside COVID-19. It ranges between 0 and 100. Source: RavenPack (https://coronavirus.ravenpack.com/)
The Insider Transactions	IT	It refers to the volume of the transactions such as purchase trades, sale trades, stock allocation, and share buybacks performed by insiders. (Source: own computation)

## Results

Table 4 reports the UQR estimates for a representative selection of quantiles.^15^ Several notable facts come to light. The value of R-squared decreases monotonically from the 10th to the 90th quantiles, indicating that the covariates diminish their importance in explaining the PIN level from the lower to the upper quantiles. Thus, the information asymmetry in a certain week is considerably more sensitive to trading volume and insider transactions during episodes when a small fraction of orders arises from private information. Interestingly, the values associated with the inferior quantiles are mostly recorded during the lockdown period, when all capital markets worldwide deviated from market efficiency (Ozkan 2021).

**Table 4:** UQR estimates—PIN—Yan and Zhang (2012) as dependent

Variables	Quantiles														
	10th			25th			50th			75th			90th		
	Model 1	Model 2	Model 3	Model 4	Model 5	Model 6	Model 7	Model 8	Model 9	Model 10	Model 11	Model 12	Model 13	Model 14	Model 15
Lagged PIN	0.011 (0.606)	0.012 (0.560)	0.015 (0.507)	− 0.087 (0.082)	− 0.086 (0.093)	− 0.081 (0.092)	− 0.159 (0.003)	− 0.166 (0.002)	− 0.164 (0.002)	− 0.152 (0.128)	− 0.164 (0.103)	− 0.170 (0.095)	− 0.079 (0.549)	− 0.086 (0.510)	− 0.083 (0.534)
Quoted Spread	0.065 (0.006)	0.064 (0.007)	0.065 (0.005)	0.064 (0.043)	0.060 (0.062)	0.062 (0.049)	0.044 (0.207)	0.054 (0.123)	0.047 (0.195)	0.152 (0.028)	0.171 (0.011)	0.157 (0.024)	0.072 (0.158)	0.076 (0.124)	0.081 (0.103)
Traded Volume	0.031 (0.000)	0.031 (0.000)	0.031 (0.000)	0.024 (0.000)	0.024 (0.000)	0.025 (0.000)	0.024 (0.000)	0.024 (0.000)	0.025 (0.000)	0.024 (0.023)	0.024 (0.022)	0.024 (0.025)	0.016 (0.069)	0.016 (0.065)	0.016 (0.056)
Market capitalization	0.002 (0.036)	0.002 (0.034)	0.002 (0.032)	− 0.001 (0.299)	− 0.001 (0.304)	− 0.001 (0.323)	− 0.001 (0.472)	− 0.001 (0.425)	− 0.001 (0.497)	0.003 (0.404)	0.002 (0.460)	0.002 (0.448)	0.001 (0.895)	0.001 (0.879)	0.001 (0.883)
Realized Volatility	− 0.061 (0.002)	− 0.066 (0.001)	− 0.071 (0.000)	− 0.047 (0.265)	− 0.060 (0.108)	− 0.069 (0.072)	− 0.088 (0.317)	− 0.074 (0.398)	− 0.089 (0.295)	− 0.158 (0.258)	− 0.130 (0.335)	− 0.133 (0.349)	− 0.203 (0.002)	− 0.181 (0.005)	− 0.184 (0.005)
Insider Transaction	− 0.001 (0.243)	− 0.001 (0.107)	− 0.001 (0.065)	0.005 (0.053)	0.005 (0.062)	0.005 (0.077)	0.009 (0.005)	0.009 (0.002)	0.009 (0.006)	0.014 (0.086)	0.015 (0.067)	0.014 (0.077)	0.040 (0.044)	0.040 (0.099)	0.030 (0.083)
Fake News	0.001 (0.306)			0.001 (0.465)			0.002 (0.361)			0.000 (0.972)			0.003 (0.349)		
Fake news *Lockdown	− 0.001 (0.740)			− 0.005 (0.492)			− 0.023 (0.035)			− 0.030 (0.096)			− 0.009 (0.582)		
Media Coverage		0.001 (0.345)			0.001 (0.348)			0.001 (0.615)			− 0.002 (0.204)			0.001 (0.458)	
Media cover *Lockdown		− 0.001 (0.620)			− 0.001 (0.563)			− 0.001 (0.000)			− 0.001 (0.030)			− 0.001 (0.174)	
Infodemic Index			0.001 (0.196)			0.001 (0.186)			0.001 (0.487)			0.001 (0.703)			0.001 (0.598)
Infodemic *Lockdown			− 0.001 (0.371)			− 0.001 (0.433)			− 0.001 (0.001)			− 0.001 (0.065)			− 0.001 (0.212)
R-squared	0.4958	0.4948	0.4934	0.299	0.238	0.2392	0.1062	0.1284	0.1105	0.082	0.0806	0.0799	0.030	0.0365	0.0369
Observation	396	396	396	396	396	396	396	396	396	396	396	396	396	396	396

We use a Gaussian kernel for the coefficients, while the robust standard errors were bootstrapped with 200 replications (Borgen 2016). The *p* values are reported in parentheses

We report a negative relationship between PIN and its previous values, which contradicts the findings of Chen et al. (2007) for NYSE. This empirical fact indicates that any sign of informed trading arising on the Bucharest Stock Exchange during a certain week is forcing other informed traders to “exit the game” in the next period. However, the estimates are statistically significant across the lower and medium quantiles, i.e., when the level of informed trading is rather moderate. Thus, the capital markets signal the possibility to earn systematic abnormal earnings based on informational asymmetry and, consequently, they quickly disappear. This result could be attributed to the architecture of the Bucharest Stock Exchange, which is a small market and the “big players”, which might possess superior information are well known.^16^ The pandemic might also cause this negative autoregressive pattern on PIN evolution, brought to light by the quantile regression methodology.

Furthermore, the trading volume has a positive and monotonically decreasing impact on PIN from inferior to superior quantiles. This result is in line with Chen and Choi (2012), but contradicts the one reported by Sankaraguruswamy et al. (2013). A possible explanation would be the lack of liquidity on the Romanian capital market, which is very low compared to developed markets. Thus, the liquidity shocks are amplifying the level of information asymmetry, especially when the latter is already low (the impact coefficient is twice as high across the 10th quantile compared to the 90th quantile). This is a normal result considering that informed traders must rely only on liquid shares to score systematic abnormal returns and to avoid at the same time the pressure of other informed traders.

The inclusion of some spread measures among the major drivers of information asymmetry proxied by PIN is justified since a longer time lag between trades causes spreads to narrow, as market makers update their beliefs. Thus, an informed trader will enter the market when the spread is relatively large, in order to optimally execute its private-information driven orders. In our case, the spread exhibits a positive impact on PIN, similar to Chen and Choi (2012). Interestingly, this result holds for inferior quantiles and for the upper ones (10th, 25th, and 75th quantiles), but not for the medium (50th quantile) and superior ones (90th quantile). Moreover, when the information asymmetry is high enough (75th quantile), the impact coefficients are almost three times larger compared to the rest of the distribution (from the 10th to 25th quantiles). Normally, the spread increases in two scenarios. First, it can be related to liquidity: if the liquidity is low, the bid-ask spread is wider. Secondly, an informed transaction, regardless of its direction (buy/sell), can impact the market because of the traded volume and might lead to a distortion in the bid or ask prices. Thus, this might lead to some reverse causality issues that need to be addressed.

Moving forward, market capitalization has an interesting impact on PIN. Across the 10th quantile, market capitalization positively impacts PIN, but the rest of the coefficients associated to higher quantiles are not statistically significant. More to the point, during episodes of reduced informed trading, an increase in the size of the company leads to an increase in the PIN. Considering the chosen period, we document that increases in market capitalizations are only determined by increases in prices. Thus, the speed or velocity of price changes, i.e., the momentum is influencing the arrival of informed traders, but only in times when the latter is reduced. This is a normal result that confirms the systematic abnormal earnings based on the informational asymmetry hypothesis mentioned previously.

In addition, volatility has a negative impact on information asymmetry (PIN), which is in line with previous studies (Sankaraguruswamy et al. 2013; Agudelo et al. 2015). However, the impact is statistically significant during extreme events of information asymmetry and not during normal market conditions.

Interestingly, COVID-19 related variables exhibit a significant impact on PIN, but only during the lockdown period, when panic is at its highest. More specifically, the impact of COVID-19 related fake news is negatively related to PIN on the Bucharest Stock Exchange, but only during the lockdown period and episodes of medium (50th quantile) or high (75th quantile) information asymmetry, confirming the first hypothesis. The same conclusion can be drawn for both media coverage and Infodemic index which validates the second stated hypothesis.

Finally, insider transactions are positively associated with PIN, which confirms the third hypothesis. This result is also valid for the majority of quantiles throughout the PIN distribution, except for the inferior ones. Compared to previous studies, this novel result directly links information asymmetry (PIN) with a measurable indicator that is not publicly available during trading sessions. In this way, we validate PIN as a predictor of the level of information asymmetry.^17^ Given the selected time window we observe a negative autoregressive pattern on PIN evolution brought to light by the quantile regression methodology.

We end this section with some robustness checks. Firstly, we use another measure for PIN, i.e. the one proposed by Gan et al. (2015). Secondly, to control for some potential endogeneity issues arising from the data, we employ the Arrelano–Bond one-step estimator (system GMM) with one lag for the dependent variable. The results are presented in Tables 5 and 6. Although the main findings mentioned previously did not change, there are some differences worth mentioning.

**Table 5:** UQR estimates—PIN Gan et al. (2015) as dependent

Variables	Quantiles														
	10th			25th			50th			75th			90th		
	Model 1	Model 2	Model 3	Model 4	Model 5	Model 6	Model 7	Model 8	Model 9	Model 10	Model 11	Model 12	Model 13	Model 14	Model 15
Lagged PIN	0.049 (0.140)	0.052 (0.121)	0.054 (0.135)	− 0.010 (0.814)	− 0.005 (0.909)	− 0.002 (0.960)	− 0.084 (0.064)	− 0.091 (0.056)	− 0.089 (0.080)	− 0.055 (0.321)	− 0.058 (0.296)	− 0.057 (0.310)	− 0.250 (0.069)	− 0.263 (0.058)	− 0.265 (0.061)
Quoted Spread	0.063 (0.022)	0.056 (0.063)	0.061 (0.031)	0.097 (0.032)	0.086 (0.067)	0.095 (0.036)	0.142 (0.004)	0.139 (0.005)	0.138 (0.003)	0.147 (0.018)	0.150 (0.013)	0.145 (0.020)	0.334 (0.096)	0.345 (0.099)	0.325 (0.137)
Traded Volume	0.020 (0.000)	0.020 (0.000)	0.021 (0.000)	0.014 (0.005)	0.015 (0.004)	0.015 (0.004)	0.019 (0.009)	0.019 (0.006)	0.020 (0.003)	0.012 (0.024)	0.012 (0.019)	0.013 (0.016)	0.017 (0.147)	0.017 (0.139)	0.017 (0.157)
Market capitalization	0.001 (0.713)	0.001 (0.635)	0.001 (0.655)	0.001 (0.495)	0.002 (0.407)	0.002 (0.430)	− 0.002 (0.368)	− 0.002 (0.373)	− 0.002 (0.386)	0.001 (0.616)	0.001 (0.647)	0.001 (0.621)	0.002 (0.563)	0.002 (0.646)	0.002 (0.590)
Realized Volatility	0.056 (0.415)	0.044 (0.540)	0.042 (0.568)	0.091 (0.011)	0.074 (0.023)	0.069 (0.042)	0.086 (0.156)	0.070 (0.210)	0.060 (0.241)	0.031 (0.645)	0.023 (0.027)	0.019 (0.786)	− 0.012 (0.961)	− 0.008 (0.971)	− 0.011 (0.961)
Insider Transaction	− 0.001 (0.080)	− 0.002 (0.047)	− 0.001 (0.055)	0.001 (0.842)	0.001 (0.971)	0.001 (0.900)	0.004 (0.000)	0.005 (0.000)	0.005 (0.000)	0.009 (0.000)	0.009 (0.000)	0.009 (0.000)	0.015 (0.089)	0.016 (0.082)	0.016 (0.091)
Fake News	0.001 (0.885)			0.001 (0.722)			− 0.001 (0.989)			0.001 (0.974)			− 0.005 (0.415)		
Fake news *Lockdown	0.003 (0.193)			0.005 (0.236)			− 0.023 (0.004)			− 0.011 (0.089)			− 0.020 (0.253)		
Media Coverage		0.001 (0.315)			0.001 (0.186)			0.001 (0.837)			− 0.001 (0.756)			− 0.003 (0.295)	
Media cover *Lockdown		0.001 (0.518)			0.001 (0.520)			− 0.001 (0.098)			− 0.001 (0.721)			0.001 (0.091)	
Infodemic Index			0.001 (0.595)			0.001 (0.343)			0.002 (0.696)			− 0.001 (0.995)			− 0.001 (0.581)
Infodemic *Lockdown			0.001 (0.784)			0.001 (0.751)			− 0.001 (0.171)			− 0.001 (0.387)			0.001 (0.898)
R-squared	0.4325	0.4357	0.4343	0.3206	0.3394	0.3341	0.146	0.1462	0.1493	0.0391	0.0322	0.0316	0.0327	0.0339	
Observation	396	396	396	396	396	396	396	396	396	396	396	396	396	396	396

We use a Gaussian kernel for the coefficients, while the robust standard errors were bootstrapped with 200 replications (Borgen 2016). The *p* values are reported in parentheses

**Table 6:** Arellano–Bond dynamic panel-data estimation

Variables	PIN—Yan and Zhang (2012) as dependent			PIN—Gan et al. (2015) as dependent		
	Model A	Model B	Model C	Model D	Model E	Model F
Lagged PIN	− 0.1560 (0.0020)	− 0.1609 (0.0010)	− 0.1640 (0.0010)	− 0.1197 (0.0160)	− 0.1352 (0.0070)	− 0.1323 (0.0090)
Quoted Spread	− 0.0433 (0.5840)	− 0.0313 (0.6900)	− 0.0379 (0.6280)	0.1131 (0.1720)	0.1303 (0.1160)	0.1147 (0.1640)
Traded Volume	0.0222 (0.0000)	0.0219 (0.0000)	0.0222 (0.0000)	0.0147 (0.0030)	0.0143 (0.0040)	0.0139 (0.0050)
Market capitalization	0.0015 (0.7540)	0.0007 (0.8770)	0.0002 (0.9590)	− 0.0060 (0.3010)	− 0.0070 (0.2210)	− 0.0074 (0.1970)
Realized Volatility	− 0.0853 (0.1900)	− 0.0653 (0.3150)	− 0.0688 (0.3000)	0.1243 (0.1090)	0.1375 (0.0770)	0.1295 (0.1050)
Insider Transaction	0.0095 (0.0020)	0.0095 (0.0020)	0.0093 (0.0030)	0.0071 (0.0570)	0.0076 (0.0410)	0.0075 (0.0450)
Fake News	0.0010 (0.6370)			− 0.0015 (0.5350)		
Fake news *Lockdown	− 0.0139 (0.0570)			− 0.0195 (0.0210)		
Media Coverage		− 0.0009 (0.1970)			− 0.0017 (0.0470)	
Media cover *Lockdown		− 0.0006 (0.0047)			− 0.0004 (0.2400)	
Infodemic Index			− 0.0003 (0.6710)			− 0.0009 (0.3150)
Infodemic *Lockdown			− 0.0006 (0.0980)			− 0.0002 (0.5930)
Observation	384	384	384	384	384	384

The *p* values are reported in parentheses

Firstly, the first-order lagged values of PIN are statistically significant only at the 50th quantile compared to the 25th and 50th quantiles in the baseline specification. The GMM estimates confirm this fact.^18^ Thus, PIN is persistent on the Bucharest Stock Exchange, but only during normal market conditions. Secondly, the market capitalization is not statistically significant under this new specification, indicating that, in the aftermath, the size is not necessarily a feature that attracts or repels informed traders. Again, similar findings are reported when using the Arrelano–Bond one-step estimator. We find similar inconsistencies associated with volatility. Thirdly, the Infodemic index is not statistically significant when PIN is estimated with the methodology proposed by Gan et al. (2015) or in the case of system GMM. The same conclusion can be drawn for the spread and media coverage.

Nevertheless, with some minor exceptions, the trading volume retains its sign and statistical significance throughout the entire distribution of PIN, suggesting that liquidity is the main driver of information asymmetry in an emerging market, especially for medium and high levels of the latter. Moreover, the higher the insider transactions, the higher the information asymmetry, especially when the level of PIN is inflated (middle and upper quantiles).

Finally, the coefficients associated with fake news during the lockdown period retain their signs, impact values, and statistical significance. This indicates that informed traders were inclined to withdraw from the market in conditions of medium or high information competition and amid the rise of COVID-19 related fake news during the lockdown period. This novel result suggests that during episodes when the level of information asymmetry proxied by PIN is medium or high (between 15 and 50%), any COVID-19 related news classified as misinformation released during the lockdown period is discouraging informed traders to place buy or sell orders conditioned by their private information. Thus, given the high uncertainty regarding the economic activity in Romania during the lockdown period (Dragoș et al. 2021), amplified by large amounts of information regarding the effects of COVID-19, any piece of private information has become redundant and vanishes.

## Conclusions

This study has explored the impact of the COVID-19 pandemic on information asymmetry, measured through PIN, for the case of the Romanian capital market. Firstly, we document that during the lockdown period, the PIN decreased. Thus, the number of orders executed based on private information decreased during the COVID-19 market crash, most likely due to increased uncertainty. Regarding factors influencing the evolution of informed-based orders, we have shown that the level of PIN depends asymmetrically on trading costs and liquidity. Furthermore, we report robust results indicating that the occurrence of insider transactions is positively influencing the information asymmetry, contributing to an increasing level of information asymmetry when looking at the medium and upper quantiles, i.e., when PIN has average or superior values, respectively. The impact coefficient is higher in absolute values across the 90th quantile compared to the 50th quantile.

Some results are in contradiction with some previous studies. This can be explained from two perspectives, namely (1) the econometric approach and (2) the sample. Regarding unconditional quantile regression, this method offers a broader view, and ultimately new insights, compared to other studies such as Aslan et al. (2011), and Sankaraguruswamy et al. (2013) on the influencing factors of PIN using a linear approach. Furthermore, our study is the first to analyse the firms listed on the Bucharest Stock Exchange, which has recently been classified as an emerging market, in a special period characterized by increased uncertainty.

These findings, especially the one linking insider trading with information asymmetry, provide a novel perspective for researchers who look for a better understanding of the price-discovery process. A slow price discovery process means that public disclosure of these trades is not sufficient to achieve price efficiency. Lastly, these results are also useful to policy makers in terms of market integrity and fairness in the capital market.

All things considered, even though this study is carried out on a single capital market, we have delivered some robust conclusions regarding the evolution of PIN and its driving factors. This approach has certain advantages in getting deeper into its specific behaviour, and might be compared to other emerging markets worldwide. A possible future direction of the study to be taken into account can be to extend the database to a panel of data during a similar uncertainty period.

Based on our results, another possible direction for future research is to find suitable strategies in an active portfolio management context, based on the premise that processing value-relevant information can lead to abnormal returns. Lastly, given the evolution of the information asymmetry and the processing needs within a pandemic context, further research can be conducted with a more pronounced focus on behavioural aspects, such as the way that different types of financial agents on the stock market address the information asymmetry throughout the evolution of the pandemic context.

## Data Availability

Data and materials will be available upon request.

## Appendix 1

See Table

**Table 7:** List of companies included in the sample

Symbol	Company	Sector
BRD	BRD—Groupe Societe Generale S.A	Financials
BVB	Bursa de Valori București S.A	Financials
COTE	Conpet S.A	Energy
EL	Societatea Energetica Electrica S.A	Utilities
M	MedLife S.A	Healthcare
OIL	Oil Terminal S.A	Energy
SNG	S.N.G.N. Romgaz S.A	Energy
SNN	Societatea Națională Nuclearelectrica S.A	Utilities
SNP	OMV Petrom S.A	Energy
TEL	C.N.T.E.E. Transelectrica S.A	Utilities
TGN	S.N.T.G.N. Transgaz S.A	Energy
TLV	Banca Transilvania S.A	Financials

*Source*: Bucharest Stock Exchange and Thomson Reuters

The Sector refers to the Economic Sector from Thomson Reuters

7.

## Appendix 2

See Table

**Table 8:** Descriptive statistics

Variable	Mean	Min	1st Quartile	Median	3rd Quartile	Max	Std. Dev
APIN	0.17	0.05	0.15	0.18	0.20	0.24	0.04
BET	8,635.81	7,342.79	8,272.17	8,650.44	8,981.19	10,138.50	634.41
ES	0.20	0.00	0.01	0.06	0.21	1.59	0.34
FNI	2.28	0.04	0.61	0.97	1.85	13.99	3.45
GAN*	0.19	0.00	0.04	0.18	0.27	0.81	0.18
GAN**	0.21	0.00	0.11	0.20	0.28	0.89	0.16
INF	48.51	11.90	40.77	46.19	62.26	78.83	17.93
LMC	21.56	18.27	20.36	21.98	22.95	23.90	1.59
LVOL	12.28	6.85	9.72	12.25	13.89	18.42	2.91
MCI	58.89	10.83	56.95	64.04	68.73	80.67	16.64
NBB	79,659.11	0.00	0.00	0.00	0.00	17,794,636.00	938,110.64
QS	0.22	0.00	0.01	0.07	0.22	1.86	0.39
RV	0.00	0.00	0.00	0.00	0.00	0.01	0.00
YZ*	0.19	0.00	0.06	0.14	0.28	0.50	0.16
YZ**	0.19	0.00	0.10	0.16	0.25	0.50	0.13

APIN—weekly PIN calculated by the average of daily values of PIN evaluated as in Cepoi and Toma (2016); *BET* BET Index, *ES* effective spread, *FNI* Fake News Index, *GAN* PIN computed using Gan et al. (2015) algorithm, *INF* the Infodemic Index, *LMC* Logarithm of the Market Cap, *LVOL* Logarithm of the Weekly Volume, *MCI* the media coverage index, *NBB* number of share buybacks, *QS* quoted spread, *RV* realized volatility, *YZ* PIN computed using Yan and Zhang (2012) algorithm

*Computed 30-min interval

**Calculated using 1-h interval

8.

## Appendix 3

See Table

**Table 9:** Trade statistics

Stocks	Average Trade Duration (minutes)	Average Trades per day	Average Trades per week*	Weekly volume	Average ASK**	Average BID*
BRD	4.7643	88	441	1,180,921.34	13.61	13.58
BVB	40.1073	4	20	3878.75	24.32	24.11
COTE	18.9323	12	60	4476.96	77.54	77.06
EL	13.6951	22	109	206,637.48	10.57	10.49
OIL	30.4341	4	21	211,560.32	18.87	18.70
M	39.0968	5	25	10,579.90	17.36	17.20
SNG	4.7032	90	448	633,345.52	31.27	31.19
SNN	6.0658	44	220	245,410.57	15.69	15.65
SNP	4.6120	95	474	44,458,800.02	0.35	0.35
TEL	14.4467	16	82	42,785.22	20.22	20.10
TGN	10.1810	22	111	7520.03	297.80	296.52
TLV	2.5356	159	797	23,149,324.54	2.25	2.25

*The average trades per week is obtained by multiplying the mean number of trades per day by 5

**The ASK and BID prices taken into consideration are not confined in the interval of time (10:15; 17:45) as the other statistics, they also reflect all the quotes not only the ones that are matched with a trade. Source: own computation

9.

## Footnotes

1 The laws of every jurisdiction stipulate the obligation of the issuers of shares that are listed on regulated markets to make public announcements about any event that is likely to impact the price of their securities. Communications must be made to the market supervisor or to the company that is managing the exchange. However, not all insider transactions concerning a specified capital market can be found or accessed for free on its corresponding national stock exchange website.

2 This is defined as the average gross profits of (potentially informed) traders (at the expense of liquidity suppliers) due to prices moving in the direction of their trading.

3 In terms of insiders’ activity, according to Romanian laws, privileged or price sensitive information represents any piece of “information of a precise nature, which has not been made public, relating directly or indirectly to one or more issuers of the financial instruments, or to one or more financial instruments and which, if it were made public, would be likely to have a significant effect on the prices of those financial instruments or on the price of related derivative financial instrument”.

4 Easley et al. (2002) and Aslan et al. (2011) compute a yearly basis PIN after excluding stocks with less than 60 days of trade and quotes data. Yan and Zhang (2012) estimate PIN on a quarterly basis for stocks that have trades and quotes available for at least 50 days in one quarter.

5 Agudelo et al (2015) use the term “dynamic PIN” when referring to the new extension of the classic PIN proposed by Easley et al. (2008). A little bit confusing, they also use this term to capture the factors that influence information asymmetry based on a panel data Tobit regression with ΔPIN_it_ as dependent variable. In our paper, we do not follow Agudelo et al (2015) since both measures of PIN that were considered as proxy for the information asymmetry—Yan and Zhang (2012) and Gan et al. (2015)—are panel stationary series. For this reason, we use level data as a dependent variable. Furthermore, we used the methods proposed by Yan and Zhang (2012) and Gan et al. (2015) instead of Easley et al. (2008) mainly due to their superiority in reducing the likelihood of boundary solutions and local maxima in MLE technique when estimating the PIN.

6 See: https://coronavirus.ravenpack.com/.

7 According to Lee and Ready (1991), the trades with a price above the midpoint are classified as buys, whilst the trades with a price below the midpoint are classified as sells. If the price equals the midpoint, then we apply a tick rule (Lee and Ready 1991). The tick rule indicates that the trade is initiated by a buyer if the price at moment *t* is above the price at the moment *t-1*, otherwise the trade is classified as a sell.

8 As in Pöppe et al. (2016), we address the issue of the overnight pause of trading and accumulated information by ignoring the trades operated at the opening hours. The Bucharest Stock Exchange is opening its continuous trading schedule at 10:00 and is closing it at 17:45.

9 We compute the PIN on a weekly basis due to the lack of liquidity on the market. Using daily data might bias the estimated results for PIN.

10 See Yan and Zhang (2012) for more details on the construction of the model and estimation of the parameters.

11 See Gan et al. (2015) for more details on the construction of the model and estimation of the parameters.

12 As we can see in Fig. 2, the differences between the two measures have become smaller during the pandemic. This fact suggests that the estimation bias is higher during normal market conditions since it is very difficult to identify all the sources of information asymmetry.

13 To have a more comprehensive view regarding PIN dynamics we present the evolution 8 months before the pandemic vs 8 months of pandemic.

14 https://bvb.ro/info/Rapoarte/Ghiduri/ghidul%20companiei%20listate%20EN%20web.pdf.

15 All the variables in UQR regression are stationary. Furthermore, we did not find any significant estimates suggesting some potential cross-sectional dependence.

16 According to the Bucharest Stock Exchange and the Financial Supervisory Authority reports (see https://m.bvb.ro/press/2022/BVB_Monthly%20report%20June%202022.pdf) in the first semester of 2022, the investment activity consisted of an average daily value of approx. 109 M RON (~ 22 M EUR) for all types of financial instruments, out of which approx. 60 M RON (~ 12 M EUR) represented the equity segment, and a total trading value of all types of financial instruments of approx. 13.5B RON (~ 2.7B EUR), out of which approx. 7.5B RON (~ 1.5B EUR) represented the equity segment. 824,000 transactions were performed by 117,000 investors present on the Romanian stock market. 20 out of the 83 issuers caring out transactions on the main market have accumulated roughly 94% of the value of the transactions made (only shares), while the top five companies accounted for approx. 64% of the total market capitalization. With respect to intermediaries, there were 27 intermediaries operating on the regulated market within the Bucharest Stock Exchange, where the top five intermediaries accounted for approx. 77% of the total amount brokered.

17 Despite its wide usage, PIN also attracts some criticism. For example, Duarte and Young (2009) suggest that PIN is not an appropriate measure of informed trading because the illiquidity component is priced. However, Brennan et al. (2015) contradicts their theory. Moreover, the studies that criticize PIN may suffer from computational and methodological biases (Ersan and Alıcı 2016).

18 To check the robustness, we compare the Arrelano–Bond estimates to 50th quantile UQR results.
